# Life or Death Decisions: Framing the Call for Help

**DOI:** 10.1371/journal.pone.0057351

**Published:** 2013-03-06

**Authors:** Eileen Y. Chou, J. Keith Murnighan

**Affiliations:** 1 Frank Batten School of Leadership and Public Policy, University of Virginia, Charlottesville, Virginia, United States of America; 2 Kellogg School of Management, Northwestern University, Evanston, Illinois, United States of America; University of Zaragoza, Spain

## Abstract

**Background:**

Chronic blood shortages in the U.S. would be alleviated by small increases, in percentage terms, of people donating blood. The current research investigated the effects of subtle changes in charity-seeking messages on the likelihood of people responses to a call for help. We predicted that “avoid losses” messages would lead to more helping behavior than “promote gains” messages would.

**Method:**

Two studies investigated the effects of message framing on helping intentions and behaviors. With the help and collaboration of the Red Cross, Study 1, a field experiment, directly assessed the effectiveness of a call for blood donations that was presented as either death-preventing (losses) or life-saving (gains), and as being of either more or less urgent need. With the help and collaboration of a local charity, Study 2, a lab experiment, assessed the effects of the gain-versus-loss framing of a donation-soliciting flyer on individuals’ expectations of others’ monetary donations as well their own volunteering behavior. Study 2 also assessed the effects of three emotional motivators - feelings of empathy, positive affect, and relational closeness.

**Result:**

Study 1 indicated that, on a college campus, describing blood donations as a way to “prevent a death” rather than “save a life” boosted the donation rate. Study 2 showed that framing a charity’s appeals as helping people to avoid a loss led to larger expected donations, increased intentions to volunteer, and more helping behavior, independent of other emotional motivators.

**Conclusion:**

This research identifies and demonstrates a reliable and effective method for increasing important helping behaviors by providing charities with concrete ideas that can effectively increase helping behavior generally and potentially death-preventing behavior in particular.

## Introduction

Each year, people donate billions of dollars to charity, as well as enough blood to prevent millions of unnecessary deaths [1]. In general, people are helpful [2]. Charities, however, have recently encountered the reality of an economic downturn, with donations in 2010 experiencing their worst drop (11%) in two decades. One of the top ten charities in the U.S., for instance, experienced a 40% drop in their donations [3].

Some charities, however, have fared better than others. Four of the top ten charities in the U.S., for instance, actually experienced increases in donations during 2010. An informal analysis indicates that the appeals of all of the six top charities that experienced donation decreases stressed their recipients’ needs for gains: “to ensure every child has a quality education” (UnitedWay Giving), “doing the most good” (Slavation Army), “to provide immediate relief and vital services” (Food for the Poor), “to further the American tradition of philanthropy” (Fidelity Charitable Gift Fund), “to continue saving lives” (American Cancer Society), and to fund “life-changing programs that help millions of children, adults and families” (The Y ). In sharp contrast, the appeals of the four top charities that experienced donation increases all focused on their recipients’ losses if help was not forthcoming: they solicited help for people “in crisis around the world” (AmeriCare Foundation), to “prevent them from going hungry” (Feed the Children), and to “reduce poverty in America” (Catholic Charities USA), which causes “more than half of the child deaths worldwide” (World Vision ). Although all ten organizations crafted their appeals in hopes of motivating the same behavior – donations – they differed markedly in their gain-loss framing. The current paper investigates the effects of this dichotomy directly. In particular, we apply the lens of prospect theory [4] to investigate whether subtle changes in charitable messages could influence people’s responses to a call for help.

A substantial stream of work has investigated the persuasive effectiveness of the content of a message as well as its framing on people’s individual choices, especially for promoting health behaviors [5]–[8]. For instance, help-seeking messages that evoke empathy, that create positive emotional feelings in the audience, or that connect them, relationally, to the recipient of their help have all been shown to increase helping behavior [9]–[17]. However, research has only rarely examined whether the framing of a help-seeking message can also influence interpersonal, helping behaviors [18]. Thus, the current research combines a well-established cognitive bias and a pressing societal predicament, as blood shortages are a recurring challenge. An increase of only 1% more of the American population giving blood every year would reduce national blood shortages to zero [1]. Thus, the effective framing of charitable messages may have the potential to substantially increase blood donations and prevent unnecessary deaths.

### Framing the Consequences: Behavioral Implications and Effects

Prospect theory suggests that the pain of losing is about twice as strong as the joy of gaining the same amount [19]. As a result, people tend to be more motivated to avoid losses than they are to achieve comparable gains [4]. Of three clear methods of framing risky choices [5], Kahneman and Tversky’s seminal paper introduced risky choice framing, i.e., altering the risks ascribed to different choice options, as a potentially potent means for changing individuals’ decisions [4]. Attribute framing, the second method, focuses on the valence of a target’s characteristics, e.g., by presenting either the success or the failure rates of different choice options [20]. Finally, goal framing focuses on the consequences of a given behavior, presenting a goal as either obtaining positive consequences or avoiding negative consequences.

The current research focuses on the effects of goal framing on helping behavior, for two reasons. First, helping behaviors do not always engender risks; and second, even if charities can report success or failure statistics, every call for help tends to have a goal. Thus, goal framing is directly pertinent to the domain of helping behaviors, as well as being practical.

The differential framing of a goal, as achieving success or avoiding failure, is also likely to have an impact on helping behavior. Research has suggested, for instance, that messages emphasizing potential losses are more effective than messages emphasizing potential gains at persuading people to engage in self-benefiting behaviors [21], especially when the stakes are high [5]. For example, women who read pamphlets that emphasized the negative consequences of not engaging in breast self-examination did more self-examinations than women who read about their positive consequences [21]. Similarly, college students who were warned about the risks of heart disease expressed stronger intentions to get cholesterol tests when the warning message was framed negatively than when it was framed positively [6].

The current research investigates whether goal framing effects can be extended from self- to other-oriented behaviors. In particular, the goal of blood donations could be described as either “to save a life” or “to prevent a death.” While the act and the consequences of donating blood remain the same, we predicted that these two messages would lead to different reactions, with seeking “avoid loss” goals leading to more helping behavior than “promote gain” goals.

Early research on helping intentions supports this prediction. Lee and Murnighan asked undergraduates to read scenarios in which a colleague was facing either a monetary gain or an equivalent monetary loss [22]. The scenarios indicated that students could intervene and help their colleague to either obtain the monetary gain or avoid the monetary loss. Self-report measures showed that help was significantly more likely when it could help to avoid a monetary loss than to obtain a monetary gain. In addition, because feelings of empathy mediated these effects, Lee and Murnighan presented their empathy-prospect model, which predicted that people would be most likely to help when feelings of empathy were strong and the target faced a loss rather than a gain (particularly a severe loss) [22].

The current research was designed to extend the model in two important ways. First, we provided a more systematic look at the framing effect by assessing potential helpers’ reactions to objectively identical messages, which also matches the kinds of predicaments that charities encounter, i.e., how to best promote a particular cause. Second, we investigated whether the differential framing of charitable messages could affect helping intentions as well as actual helping behaviors. Because helping intentions do not always translate into helping behavior [23], the practical value of successfully encouraging actual helping behavior may be especially significant.

### The Current Research

We present two studies that investigated the effects of loss framing on helping intentions and behaviors. With the help and collaboration of the Red Cross, Study 1, a field experiment, directly assessed the effectiveness of a call for blood donations that was presented as either death-preventing (losses) or life-saving (gains), and as being of either more or less urgent need. With the help and collaboration of a local charity, Study 2, a lab experiment, assessed the effects of the gain-versus-loss framing of a donation-soliciting flyer on individuals’ expectations of others’ monetary donations as well their own volunteering behavior. Study 2 also tested the robustness of the loss framing effect by assessing its independence from other emotional motivators. Together, the results not only extend the theoretical implications of message framing, they also create a new, action-oriented imperative for helping behavior.

### Study 1

Study 1 also investigated people’s behavioral reactions to the gain- or loss-framing of an opportunity to donate blood when a target’s needs were characterized as either urgent or not. Lee and Murnighan found, not surprisingly, that the prospect of more versus less severe losses led to stronger helping intentions [22]. The current study investigates the impact of severity in terms of urgency, as urgency is particularly relevant for blood donations. For instance, in the week following September 11th, 2001, U.S. blood centers received 2.5 times more blood than they did for the same week the previous year [24]. Thus, we predicted that the potential for urgent losses would lead to more donations than either gain-frames or less urgent needs, as the empathy-prospect model [22] would predict.

To test this prediction, we created five email messages to advertise a blood drive. A control condition message included only basic information about the time and the place for people to donate. Four additional conditions included this same information and described the blood drive as either life-saving or death-avoiding and the need as either urgent or moderate.

## Method

### 

#### Participants, design, and procedure

We obtained IRB approval from the Northwestern University IRB review board and the consent of LifeSource Blood Center to conduct this research. Because of the nonintrusive nature of the study design and the fact that the data were analyzed anonymously, the review board waived the need to obtain the participants’ written consent.

Two days prior to the event, we emailed information about a blood drive to 3534 undergraduates in 19 residence groups (ten dorms and nine sororities and fraternities. We targeted these residence groups to ensure that each of them would only receive one email message). We divided the 19 groups into five separate clusters of relatively equal size (*M* = 706.80, *SD* = 75.67). The control condition emails only contained time and location information. Each of the other four clusters received emails that also described blood donations as either life-saving or death-avoiding, and of urgent or moderate need. This extra information read as follows, with the gain-oriented text in parenthesis, and the less urgent text in italics:

“Don’t delay. Help prevent someone from dying! (Act now. Help save someone’s life!) Each year, 4.5 million Americans would die without blood transfusions. Every second (*every day*), 2 (*many*) people could die waiting for (can be saved by donated) blood … Every pint that you donate can help them avoid dying (stay healthy) … Don’t delay (Act now) … help prevent unnecessary deaths (promote healthy lives).”

Although not everyone who came to the blood drive passed the physical exam required to donate, everyone who showed up was counted as a donor. Each donor received a survey asking them to identify their campus residence and whether they belonged to a fraternity or sorority; this information allowed us to identify which email message they had received.

### Results and Discussion

**Figure 1:**
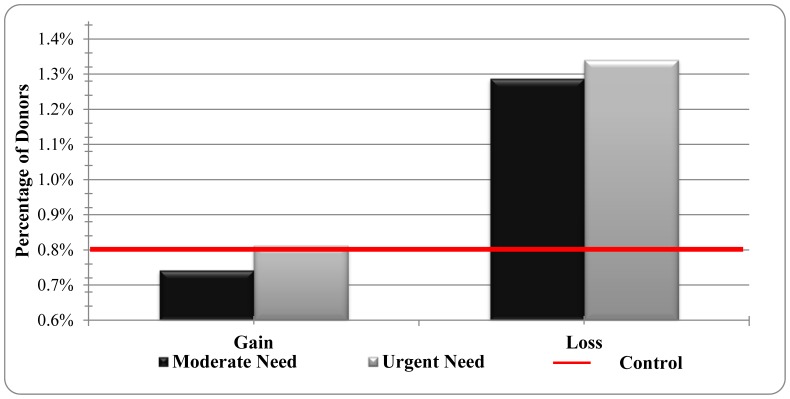
Blood donor percentages by condition.

Over the two days of the blood drive, 119 people showed up to donate blood. Nonparametric analysis indicated that ‘Prevent a death’ emails led to a significantly higher donor rate (1.31%) than ‘Save a life’ (0.78%; *χ^2^*(1) = 5.40, *p* = 0.02) or control emails (0.80%; *χ^2^*(1) = 4.87, *p* = 0.02), which did not differ from each other (*χ^2^*(1) = 0.01; *p* = 0.99). In addition, emails indicating ‘urgent needs’ (1.09%) did not lead to more donors than ‘moderate needs’ (1.00%; *χ^2^*(1) = .13, *p* = .72) or control emails (0.80%; *χ^2^*(1) = 1.66; *p* = 0.19). Logistic regression analysis also indicated that the interaction between framing and urgency was not significant (*B* = -.05, *SE* = .76, *χ^2^*(1) = 0.01; *p* = 0.94; see Figure 1).

These results indicate that a simple change in the framing of an emailed request, from saving a life to preventing a death, led to a significant increase in the blood donation rate. Moreover, this effect had its impact after a two-day delay and did not depend on levels of urgency. Recent data suggests that only 37.8% of the US population is eligible to donate blood but less than 10% of this group donate blood at least once a year [25]. Thus, these findings suggest that the phrasing of blood donation entreaties may be a fruitful and cost-effective way to obtain more blood donations.

Results from Study 1 directly demonstrated the impact that a loss-framing message can have on inducing helping behaviors. Study 2 was designed to determine whether three emotional antecedents to helping behaviors have an impact on this effect.

### Study 2

Charitable organizations have often crafted their charitable messages to be as emotionally captivating as possible. Indeed, past research has identified various emotional factors that lead people to be more helpful [9]–[11]. Study 2 investigates whether three theoretically-grounded antecedents of helping behavior - feelings of empathy [12], positive affect [13]–[14], and the relational closeness of the target [10], [15]–[16] – would influence the message framing effect.

Empathy, defined as “an other-oriented emotional response elicited by and congruent with the perceived welfare of another” (p.285), often leads to increases in helping behavior [9]. In particular, data on the empathy-altruism model [9], [12], [26] depict a process in which a potential helper sees someone in need, feels a sense of empathy toward them, and the strength of these feelings determine how much they help. Although the empathy-altruism model has been attacked [10], [14], research has consistently shown that felt empathy is positively related to helping behavior, in a variety of contexts. For example, people who felt more empathy toward others have cooperated in prisoner’s dilemma games [9], shared their class notes [27], volunteered to endure physical pain on another’s behalf [12], and indicated that they would accept lesser monetary gains [22].

Other theoretical approaches to helping behavior focus on helpers’ positive affect, predicting that people in a positive mood will be more likely to help [11], [13], [28]. Positive mood augments helping behavior when the helping tasks are inherently rewarding and can foster subsequent positive affect [13], [29]. In addition, when the benefits of helping are high and cost is low, positive mood states have led to more helping behavior than neutral mood states [30].

A third approach suggests that people will help more when they have a closer relationship with the target [10], [14]. Thus, people prefer to help friends more than acquaintances [10], family members more than strangers [15], [16], and ingroup more than outgroup members [31].

Study 2 assessed whether these three factors had an impact on the positive effects of loss-framing. In Study 2 we collaborated with a local charity to create two donation-soliciting flyers that framed the charity’s goals as either increasing or preventing a decline in their effectiveness. Participants were randomly assigned to read and evaluate one of the two flyers and then indicated their willingness to help; they also engaged in voluntary helping behavior, as well as reporting their feelings of empathy, positive affect, and relational closeness. We expected that the loss-framed flyer would lead to more help than the gain-framed flyer would; whether the three emotional factors might influence this effect was an open question.

## Method

### 

#### Participants and design

We obtained IRB approval from the Northwestern University IRB review board to conduct this research. Participants were 182 undergraduates (63.8% female) at a Northwestern University, recruited from the business school’s subject pool; they provided informed written consent to be included in this study and received $8 for participating. We manipulated the gain-loss framing of the flyer in a one-factor between-subjects design.

#### Procedure

After arriving at the laboratory, participants were asked to read about a nearby nonprofit organization that aims to “assist low-income individuals and families…to stabilize their lives and develop the skills necessary to become productive community members.”They then learned that the organization was seeking feedback on its new flyers, which described the charity’s programs and their current performance. The flyers also described John, a recently unemployed construction worker who was enrolled in their workforce assistance program. (Although John was typical of many people who received help from this charity, he did not actually exist.).

We manipulated the flyer’s request for help by indicating that contributors could either “minimize the possibility of a decrease” or “maximize the possibility of an increase” in the charity’s ability to help John and others get “out of unemployment” or “into new jobs.” Participants then evaluated the flyer’s effectiveness by indicating how much money they thought that a philanthropist who saw this pamphlet would donate to help the center and people like John (from $0 to $100,000). Because the amounts varied widely, we converted these responses by log transforming them prior to analysis. (We report the means of the unstandardized means in the text for ease of interpretation). Participants then responded to single items asking them how empathetic and happy they felt, on 7-point scales (1 = *not at all* to 7 = *very much so*), and two items indicating how much they felt that they knew John or someone similar to John, on 3-point scales (1 = *not at all* to 3 = *very well*). We averaged these last two items, which were highly correlated (r = .92), to form a Relational Closeness scale.

Participants then learned that they could help John and the charity by preparing letterheads for his job search. The instructions stressed that their payment for participating in the experiment would not be affected by their willingness to help and that helping was completely voluntary: they could work on as many or as few letterheads as they wished and they could stop at any time. Participants who indicated a willingness to help received a packet containing detailed instructions; non-volunteers were asked to stay in their cubicles for the remaining time of the session. (Many of them used the computer in their cubicle to surf the internet.).

Volunteers were directed to a word document containing the template of a letter that John had written and a list of 26 organizations that had contributed to the job fair. Their task was to create separate letterheads for each organization, using John’s template. This required manual entry of the name of an organization and its address in the header of each letter. Thus, participants could not copy and paste one document into the other. We counted how many letters they completed; scores ranged from zero to 26. An expressed willingness to help was used as an indicator of helping intentions; actual amount helped, i.e., number of letterheads prepared, was our measure of helping behavior. All of the participants, regardless of whether they volunteered or not, stayed for an additional 10 minutes before they were thanked, paid, and excused.

### Results and Discussion

As expected, the loss frame led to more expected donations (*M* = $12,425.57, *SD = *$20,956.39) than the gain frame (*M* = $7,019.38, *SD = *$10,601.58), *t* (170) = 2.73, *p* = 0.007.

We also conducted a heteroskedasticity-consistent standard error estimator to address potential heteroscedasticity concerns [32]. This test used a standard error estimator that does not assume homoscedasticity. Results from this robust inference test confirmed that loss framing led to more expected donations than gain framing did, *F* (1, 180) = 7.46, *p* = 0.007.The gain and loss frames, however, did not affect participants’ self-reported empathy, positive affect, or relational closeness, *t* (180) <1, *p*>0.47, *d* <.03 for all three. (Table 1 presents the descriptive statistics and zero-order correlations of the variables.).

**Table 1:** Means, standard deviations, and correlations of the variables.

Variable	*M*	*SD*	1	2	3	4	5
1. Expected Donations	9,633.3	16,618.7					
2. Volunteer	0.56	0.49	−0.1				
3. Letterheads Completed	1.17	3.87	−0.04	.27*			
4. Felt Empathy	4.11	1.56	.21*	−0.02	−0.07		
5. Reported Happiness	4.7	1.09	0.12	−0.01	0.13	.26*	
6. Relational Closeness	0.73	0.8	−0.05	−0.14	−0.05	−0.07	0.01

*Note:* Volunteer was a dichotomous variable (Yes = 1; No = 0); Letterheads completed ranged in number from 1 to 26; Felt Empathy and Reported Happiness were self-reported on 7-point scales, with 7 indicating greater empathy and happiness; and Close Relations was a 2-item index, with from 1 = not at all to 3 = very well.

* *p*<.01.

We used multiple regression analyses to assess whether the gain-loss effects on expected donations were influenced by individuals’ self-reported emotions and relational closeness (see Table 2). Model 1 shows that framing the charity’s request as a potential loss was associated with higher expected donations than framing it as a potential gain (*B* = .32, *SE* = .14, *p* = .02). Model 2 shows the effects of felt-empathy, positive affect, and relationship closeness on expected donations. Perceived empathy, on its own, was significantly associated with higher expected donations, but positive affect and relational closeness were not. In addition, Model 3 shows that gain-loss framing remained significant after accounting for the effects of all three emotional motivators (ΔR^2^ = .03, *p* = .03). This pattern of effects was consistent for all possible orders of inputting these four variables.

**Table 2:** The results of linear regression models on expected donations.

	Model 1	Model 2	Model 3
Variable	β	β	β
Framing	.16*	–	.16*
Felt Empathy	–	.19*	.19*
Reported Happiness	–	.07	.07
Relational Closeness	–	−.03	−.03
Adjusted R^2^	.02	.03	.05
F-value	4.91*	3.16*	3.61**

*Note:* Framing effect: 0 =  Gain and 1 =  Loss.

* *p*<.05,

** *p*<.01.

**Figure 2:**
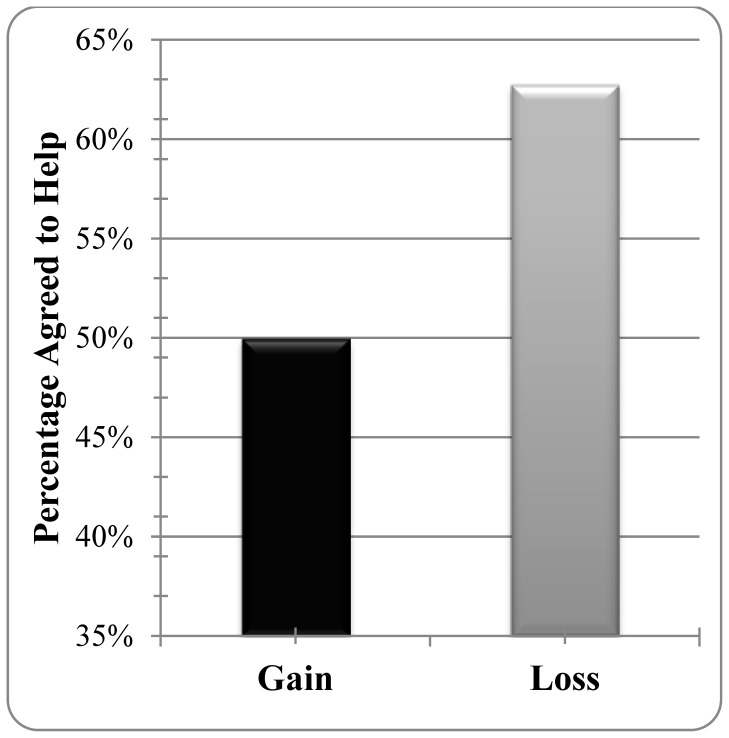
Percentage of participants who indicated that they would help as a function of the gain/loss framing of the charity’s request.

We then analyzed the same four variables’ effects on people’s volunteering intentions. A binary logistic regression analysis showed that the loss-framed flyer led to a marginally higher percentage of people (60.2%) who agreed to volunteer than the gain-framed flyer did (52.1%; *B* = .54, *SE* = .30. *χ^2^*(1) = 3.12, *p = *.07; see Figure 2). The gain-loss effects on volunteering were not influenced by individuals’ self-reported empathy or positive affect (*χ^2^*(1) <1, *p*>.76). Unlike previous research [10], the regression results indicated that relational closeness was associated with less volunteering (*B* = -.37, *SE* = .19, *χ^2^*(1) = 3.69, *p* = .05).

**Figure 3:**
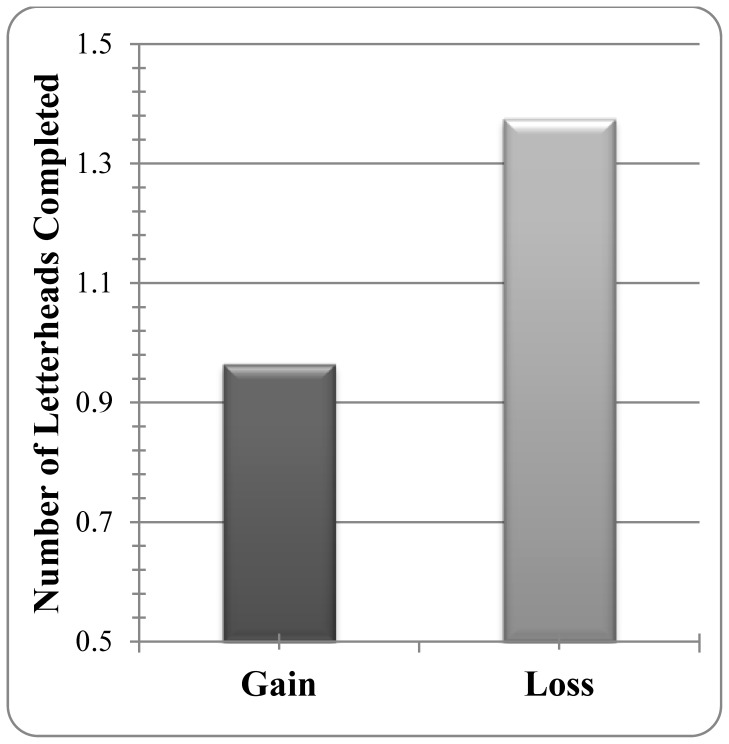
The mean number of letterheads completed per volunteer as a function of the gain/loss framing of the charity’s request.

Although over half of our participants volunteered to help, only 18.63% of those who volunteered to help actually engaged in helping behaviors. A Poisson regression showed that those who did help prepared significantly more letterheads for the loss- rather than the gain-framed flyer (*B* = .35, *SE* = .14. *χ^2^*(1) = 6.37, *p = *.01; see Figure 3). This low rate of actual volunteering is consistent with previous findings showing that, as costs increase, people help less [30], [33]. More importantly, these findings show that framing a goal as avoiding a potential loss again had stronger behavioral effects on actual helping behavior than framing it as achieving a gain. The data also show that three emotional motivators that have led to increased helping behavior in previous research did not interfere with or limit loss framing’s significant effect.

## General Discussion

The current research has shown how subtle changes in charitable messages can have profound effects on how people respond to a call for help, and that these effects act in addition to the positive impact of other emotional factors that also motivate helping. In both of the studies reported here, framing a goal as either a potential gain or a potential loss influenced interpersonal helping behavior. Study 1 showed that significantly more donors volunteered to give blood when the message soliciting their participation was framed as death-preventing rather than as life-saving, and the loss flyers in Study 2 led people to predict larger monetary donations, increased their willingness to volunteer, and led them to help more, independent of felt-empathy, positive affect, and relational closeness.

A central issue in the helping literature often contrasts two underlying reasons for helping. Pure altruism, i.e., the selfless desire to improve the welfare of others [12], is conceptually different from impure altruism, such as helping behaviors that are motivated by self-regarding desires (e.g., to alleviate a person’s own psychological discomfort from not helping) [9], [10], [12], [28]. Rather than engaging in the philosophical debate on whether pure altruism exists, the current studies attempted to accommodate both pure and impure altruism by focusing on the cognitive factors that can directly affect individuals’ helping decisions. The data show that goal framing’s effects on helping were not contingent on whether the motivation was either self or other regarding. Instead, the data suggest that a primarily cognitive rather than an emotional approach can have a potent effect on a helper’s decision processes.

These results also provide behavioral support for the empathy-prospect model [22], showing that the description of a helping opportunity as a chance to help someone avoid a loss leads to more helping behavior than the description of the same opportunity as a chance to help someone achieve a gain. Although our severity manipulation did not have an effect in Study 1, hindsight suggests that the differences in our two conditions was not particularly strong. Alternatively, the current findings might have reflected a ceiling effect in how people reacted to severity. Thus, we encourage future research to better detect severity’s effects.

In addition, past research has shown that people tend to process negative information longer and more deeply [5], [34] and they weigh it more heavily than positive information [35]. Thus, in the current context, as people consider the consequences of not helping, walking away may become more difficult, independent of other emotional effects. These ideas also provide fertile ground for future research.

### Conclusions

To help another person (or not) is among the most important and consequential decisions that an individual can make. By extending prospect theory from individually- to interpersonally-relevant behavior, we found that others’ losses and gains significantly affected individuals’ actual behavior in the same way that a person’s own losses and gains do. Thus, by taking advantage of the biasing properties of a loss frame, charities may be able to significantly increase important helping behaviors. Our hope is that it can provide a variety of concrete ideas that can effectively increase helping behavior generally and potentially death-preventing behavior in particular.
